# Improving Molecule–Metal Surface Reaction Networks Using the Meta-Generalized Gradient Approximation: CO_2_ Hydrogenation

**DOI:** 10.1021/acs.jpcc.4c01110

**Published:** 2024-05-17

**Authors:** Yuxiang Cai, Roel Michiels, Federica De Luca, Erik Neyts, Xin Tu, Annemie Bogaerts, Nick Gerrits

**Affiliations:** † Research Group PLASMANT, Department of Chemistry, University of Antwerp, Universiteitsplein 1, Antwerp, Wilrijk BE-2610, Belgium; ‡ Department of Electrical Engineering and Electronics, University of Liverpool, Liverpool L69 3GJ, U.K.; § Department of ChiBioFarAM (Industrial Chemistry), ERIC aisbl and INSTM/CASPE, University of Messina, V.le F. Stagno d’Alcontres 31, Messina 98166, Italy; ∥ Leiden Institute of Chemistry, Gorlaeus Laboratories, Leiden University, P.O. Box 9502, Leiden 2300 RA, The Netherlands

## Abstract

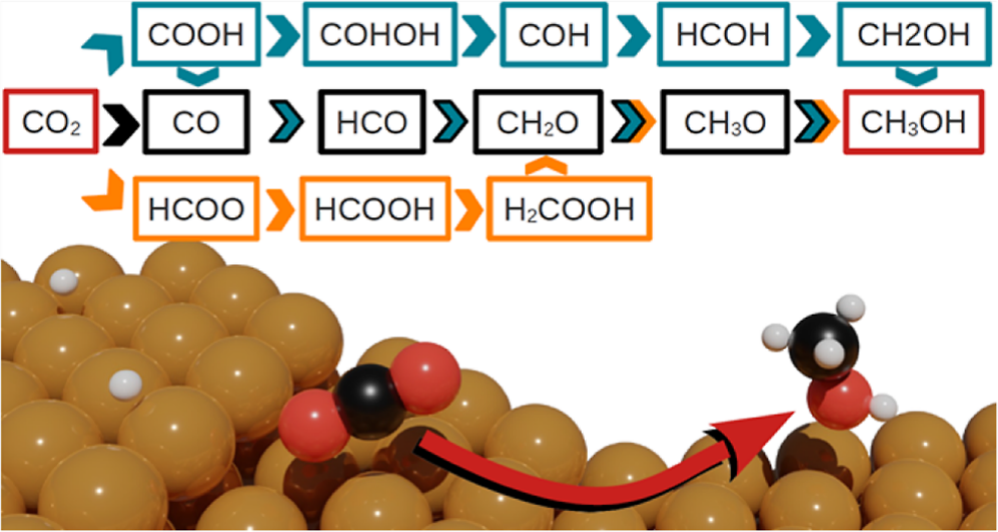

Density functional theory is widely used to gain insights into molecule–metal surface reaction networks, which is important for a better understanding of catalysis. However, it is well-known that generalized gradient approximation (GGA) density functionals (DFs), most often used for the study of reaction networks, struggle to correctly describe both gas-phase molecules and metal surfaces. Also, GGA DFs typically underestimate reaction barriers due to an underestimation of the self-interaction energy. Screened hybrid GGA DFs have been shown to reduce this problem but are currently intractable for wide usage. In this work, we use a more affordable meta-GGA (mGGA) DF in combination with a nonlocal correlation DF for the first time to study and gain new insights into a catalytically important surface reaction network, namely, CO_2_ hydrogenation on Cu. We show that the mGGA DF used, namely, rMS-RPBEl-rVV10, outperforms typical GGA DFs by providing similar or better predictions for metals and molecules, as well as molecule–metal surface adsorption and activation energies. Hence, it is a better choice for constructing molecule–metal surface reaction networks.

## 1 Introduction

Climate change is one of the most important challenges humanity faces in the 21st century. To limit global warming, it is essential to reduce carbon dioxide (CO_2_) emissions.^1^ The catalytic hydrogenation of CO_2_ to methanol (CH_3_OH) is of particular interest as CH_3_OH is considered both a clean fuel and a versatile feedstock for green chemistry.^2^ Despite the industrialization of this technology, the underlying mechanism remains poorly understood.^2^ A common challenge pertains to the identification of the reaction sites and the carbon species’ evolution in the reaction network. The latter bears critical implications for the development of novel CO_2_ hydrogenation processes, such as electrocatalysis and plasma catalysis.

Density functional theory (DFT) has been widely used to gain insights into the energetics and mechanisms of catalytic reactions on surfaces. Moreover, the results of such DFT studies can also be used in kinetic and multiscale modeling of catalytic reactions. However, it is difficult to determine the exact surface mechanism of most catalytic reactions using DFT. This is, in part, due to the dependency of the results on the choice of density functional (DF).^3^ Most studies use generalized gradient approximation (GGA) DFs, i.e., DFs that only depend on the electron density and its gradient. However, GGA DFs are known to struggle to simultaneously provide accurate predictions of both molecular gas-phase and metal surface energies,^4^ where both are crucial for the study of reactions at metal surfaces. Specifically, GGA DFs that excel at describing adsorption energies tend to underestimate the energy of the metal surface itself and overestimate the metal lattice constant, while GGA DFs that perform well for metals tend to overestimate adsorption energies.^5^ Furthermore, GGA DFs tend to systematically underestimate barrier heights.^6^ To address these limitations, meta-GGA (mGGA) DFs introduce a dependency on the kinetic energy density (KED). This allows them to distinguish between regions with electron densities describing molecular orbitals, metallic orbitals, and weak bonds, resulting in a DF that can accurately describe both metal surfaces and gas-phase molecules. Several mGGA DFs^7−10^ also have a hydrogen self-interaction error (SIE) correction, i.e., a parameter is introduced that is fitted so the DF reproduces the exact exchange energy of a free hydrogen atom. This yields an approximate correction in the molecular orbital regime to the SIE inherent to DFT. This interaction of an electron with itself arises due to the use of the classical expression for the Coulomb interaction of electron densities. The introduction of the KED also allows us to satisfy additional theoretical constraints compared to GGA DFs.^4,10,11^ Besides the choice of DF, when studying surface reaction networks, it can also be important to explicitly compute reaction barriers as the use of scaling relations can be problematic. For example, it has been found that the SIE tends to be lower for adsorption than for the transition state (TS),^6^ meaning that the DF behaves differently for the calculation of adsorption energies and activation energy barriers, necessitating the explicit calculation of the barrier.^12^ Lastly, it is vital to comprehensively study the reaction network as a whole and not one possible mechanism separately as this leads to the a priori exclusion of other mechanisms. In this work, we will study a surface reaction network for the first time using a mGGA DF and illustrate that the mGGA DF outperforms GGA DFs in the description of the system, namely, CO_2_ hydrogenation toward CH_3_OH over a Cu surface.

**Figure 1:**
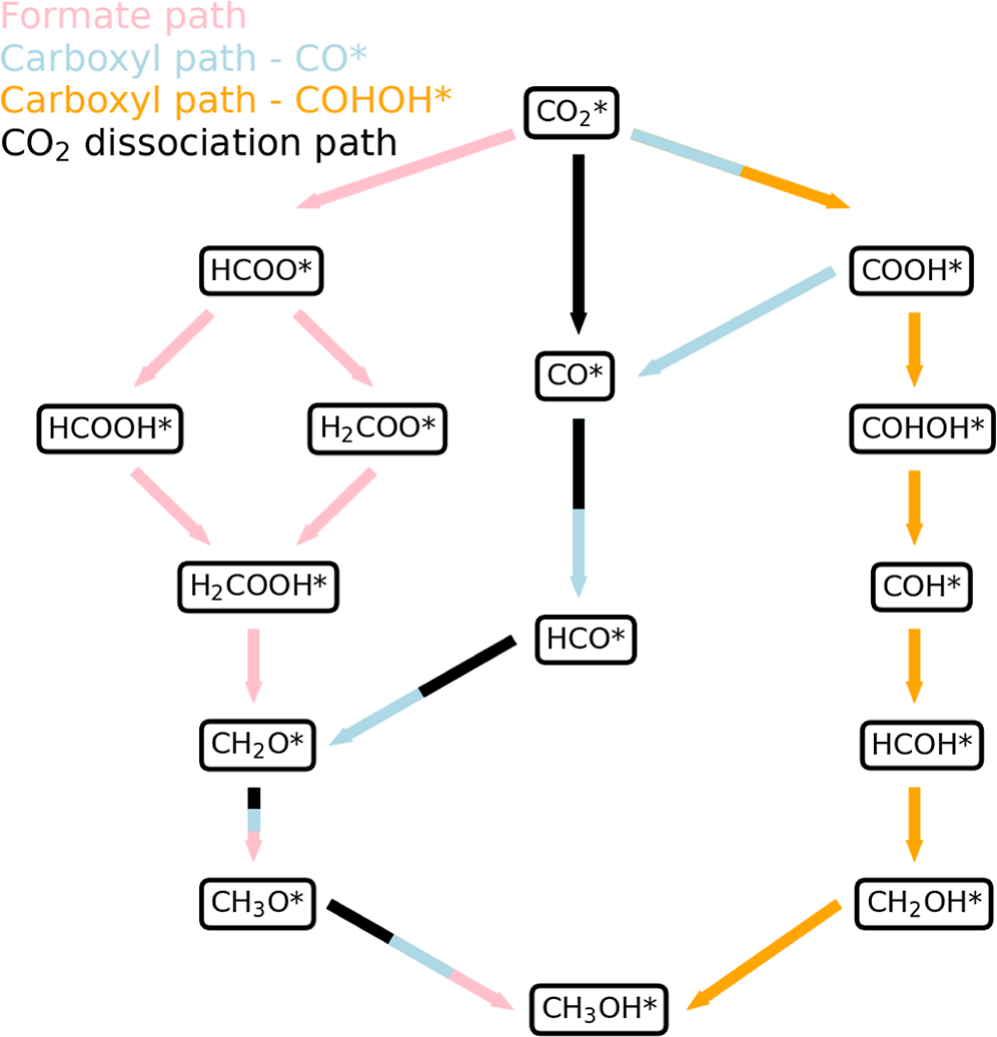
Overview of possible pathways from CO_2_ to CH_3_OH on Cu(111) and Cu(211) surfaces. The formate path is indicated in pink, the carboxyl path through CO* in blue, the carboxyl path through COHOH* in orange, and the CO_2_ dissociation path in black.

Several DFT studies on the reaction mechanism of CO_2_ hydrogenation toward CH_3_OH on Cu were previously carried out.^13−20^ The active site for this reaction is still unclear, so we will discuss both the flat Cu(111) and stepped Cu(211) surfaces as the possible pathways investigated in literature are the same for both facets. All possible pathways are depicted in a reaction network in Figure 1. In the formate pathway, in pink, CO_2_* (* denotes adsorbed species) is hydrogenated to HCOO* and subsequently either to HCOOH* or H_2_COO*. In the carboxyl pathway, CO_2_* is hydrogenated to COOH*, which can either dissociate to CO* and OH*, in blue, or be hydrogenated to COHOH*, in orange. Finally, in the CO_2_ dissociation pathway, in black, CO_2_* dissociates into CO* and O* without going through another intermediate first. It is worth noting that most of the aforementioned studies did not comprehensively investigate all reaction pathways mentioned above simultaneously but rather focused on one or a few possible reaction mechanisms. Furthermore, all of them employed a GGA DF.

Studt et al.^21^ compared two GGA DFs, i.e., BEEF-vdW^22^ and revised Perdew–Burke–Ernzerhof (RPBE),^23^ for the description of CO_2_ hydrogenation on Cu(211). They concluded that BEEF-vdW improves the description of CH_3_OH synthesis and partially attributed this to the inclusion of long-range correlation effects in the BEEF-vdW DF, which are missing in the semilocal RPBE DF. However, it is important to note that they apply an empirical correction to the energies of species containing an OCO backbone. It is unclear to us to what degree these corrections are responsible for the improved performance of BEEF-vdW. Nevertheless, the inclusion of long-range nonlocal correlation and the importance of van der Waals forces have also been suggested by Garza et al.^24^ to improve the description of chemisorption on metal surfaces, also when using a mGGA DF.

It is clear that for a good description of reaction networks on metal surfaces, a mGGA DF combined with the inclusion of long-range correlation is needed. One way to construct a mGGA DF is by introducing an inhomogeneity parameter α associated with the KED, which allows the DF to distinguish between the different density regimes associated with the metal surface and the gas-phase molecule. Unfortunately, the choice of mGGA DF is also not trivial. For example, the strongly constrained and appropriately normed (SCAN)^8^ DF has been shown to greatly underestimate molecule–metal surface reaction barrier heights.^10,25^ A recent development in this field is the made-simple (MS) DFs.^7,10,26^ In these DFs, an interpolation function dependent on α is used to switch between the metallic and atomic regimes, where a correction to the atomic regime is made to reproduce the exact exchange energy of a free hydrogen atom.

The first MS DF, MS0,^26^ was found to outperform the standard PBE^27^ GGA DF in predicting exchange energies of rare gas atoms, atomization energies, enthalpies of formation, and lattice constants. It was found to perform similarly to the revTPSS^28^ mGGA. MS0^26^ was later parameterized by including one or two empirical parameters, resulting in the MS1 and MS2 DFs, respectively.^7^ Overall, MS1 and MS2 were found to improve over MS0, and all MS DFs showed robust performance in predicting heats of formation, barrier heights, and weak interactions.^7^ The performance of these MS DFs was compared to that of GGA DFs, including PBE,^27^ and to that of mGGA DFs, including M06-L^29^ and revTPSS.^28^ MS1 was found to perform best in predicting a set of heats of formation, while M06-L was found to perform best in predicting a set of barriers, closely followed by MS0, MS1, and MS2.^29^ The authors noted that the number of parameters in M06-L is an order of magnitude larger than the number of parameters in the MS DFs.^29^ These results show that the MS DFs are robust and perform well in describing gas-phase molecules.

Smeets et al.^10^ recently proposed three MS mGGA DFs (MS-PBEl, MS-B86bl, and MS-RPBEl) based on the exchange expressions of PBE,^27^ B86b,^30^ and RPBE^23^ but with the exchange gradient expansion coefficient μ taken from PBEsol.^31^ The GGA correlation expression was taken from revTPSS.^28^ They studied the decomposition of H_2_ on Cu(111) and Ag(111) surfaces and found that these DFs exhibit similar accuracy to PBEsol in predicting the properties of metallic Cu and Ag while outperforming both the GGA DFs PBE and RPBE and mGGA DFs SCAN^8^ and TPSS^9^ in predicting experimental molecular beam sticking probabilities on Cu(111). Furthermore, they found that DFs excellently predicted the experimental interlayer lattice spacing for Cu and Ag and the lattice parameter for Pt and Au. Recently, the performance of MS-B86bl was investigated in the SBH17 benchmarking study, a database for dissociative chemisorption barrier heights.^25^ It was found that the DF performed well for predicting metal properties. Moreover, for 16 of the 17 studied dissociative chemisorption systems, MS-B86bl yielded accurate saddle point geometries and a mean absolute error of 0.17 eV for the dissociation barrier heights, only failing to obtain a self-consistent saddle point for H_2_ + Pt(211), which is a very shallow barrier and difficult to obtain with both GGA and mGGA DFs. Although MS-B86bl was found to be a somewhat mediocre DF compared to other (m)GGA DFs for SBH17, it should also be noted that SBH17 only contains reactions for which GGA DFs excel^6,25^ and that MS-B86bl lacks long-range correlation. For example, MS-RPBEl has also been used to describe dissociative chemisorption of O_2_ on Al(111)^6^ and HCl on Au(111),^32^ two infamous examples where all GGA DFs severely overestimate the reactivity. In general, they yielded considerably improved agreement with experimental sticking and inelastic scattering probabilities compared to GGA DFs. Moreover, in a later study, Smeets and Kroes^33^ combined these MS mGGA DFs with rVV10,^34^ a self-consistent nonlocal correlation DF. As mentioned above, the inclusion of nonlocal correlation is critical for a good description of reaction networks on metal surfaces. The inclusion of rVV10 slightly reduced the accuracy of the metal description but improved the description of D_2_ dissociative adsorption on Ag(111), Pt(111), and Au(111). MS-PBEl-rVV10 was later used to study CHD_3_ dissociation on a Pt(110)-(2 × 1) surface and led to good agreement with experimental results.^35^ The above shows that the combination of a high-accuracy mGGA DF with rVV10 overcomes the limitations of GGA DFs by solving the challenges of the description in metal surface layers and of the molecule and including long-range interactions, critical when describing adsorption.^21,24^ Moreover, the computational cost of a mGGA DF is roughly a factor three more than that of a GGA DF, making it a cost-effective option for constructing comprehensive and self-consistent reaction networks.

Here, we use a regularization of the inhomogeneity parameter α, which is used in most mGGA DFs, that was introduced in r^2^SCAN.^36^ This regularization improved the numerical performance of the SCAN functional while maintaining its accuracy, which is observed here as well, i.e., the regularization does not affect the results. Note that the regularized parameter is only used in the exchange part of the DF as revTPSS GGA correlation is employed in the MS mGGA DFs, which does not contain the α parameter.

To the best of our knowledge, no mGGA DF has so far been applied for the study of a reaction network on any metal surface. Hence, in this work, we will use an MS mGGA DF, i.e., regularized MS-RPBEl, combined with rVV10 nonlocal correlation (rMS-RPBEl-rVV10), to study CO_2_ hydrogenation on Cu(111) and Cu(211). We hope, and indeed conclude, that this will lead to a better understanding due to the improved description of both the metal and molecule by the MS mGGA DFs and the inclusion of nonlocal correlation via rVV10. To evaluate the performance of the rMS-RPBEl-rVV10 DF on this reaction network, three GGA-level DFs with vdW correction, namely, PBE-D3,^27,37^ RPBE-D3,^23,37^ and BEEF-vdW^22^ were chosen for comparison.

## 2 Methods

Periodic plane-wave DFT calculations were carried out using the Vienna ab initio simulation package (VASP, version 6.2.1).^38−43^ The regularized MS RPBE-like (rMS-RPBEl)^10^ mGGA DF was used in combination with the rVV10^34^ DF to account for nonlocal correlation effects. This gives the following expression for the exchange–correlation functional^33^
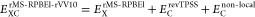
where *E*_X_^rMS-RPBEl^ is the MS-RPBEl exchange DF, *E*_C_^revTPSS^ is the semilocal revTPSS^28^ GGA correlation DF (i.e., GGA instead of mGGA correlation is employed), and *E*_C_^non-local^ is the non-local rVV10^34^ correlation DF. For a detailed explanation on the latter two parts of the DF, we refer the reader to the respective references. *E*_X_^rMS-RPBEl^ is given by^26^
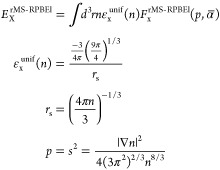
Here, *n* is the density and ε_x_^unif^ is the exchange energy per particle of a uniform electron gas (UEG). The exchange enhancement factor *F*_x_^rMS-RPBEl^ is given by ref (33) as
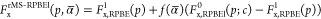


The exchange enhancement factor is used to obtain the exchange part of the exchange–correlation energy by interpolating between two extreme cases: the UEG and a single-orbital system. *F*_x,RPBE_^1^(*p*) and *F*_x,RPBE_^0^(*p*;*c*) are the gradient-only-dependent exchange enhancement factors for the UEG and single-orbital cases, respectively. The former is expressed as
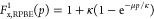


For *F*_x,RPBE_^0^, *μp* is replaced in the formula above by *μp* + *c*. *μ* is 10/81, *κ* is 0.804, and *c* is 0.07671. For a more detailed explanation of the parameter values, we refer the reader to the publication by Smeets et al.^10^ The interpolation function *f*() depends on the KED τ through the inhomogeneity parameter α^10,36^
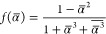

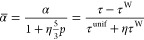
where τ^W^ is the von Weizsäcker KED, τ^unif^ is the KED of the UEG, and *η* is a regularization parameter equal to 0.001. Expressions for these KEDs can be found in the paper by Sun et al.^26^ In the single-orbital case, which is a good model for covalent bonding,  = 0 because τ^W^ = τ, and thus *f*() = 1. This leads to the exchange energy being determined only by the single-orbital exchange enhancement factor *F*_x_^0^. In the UEG case, which is a good model for metallic bonding,  approaches 1 because τ ≈ τ^unif^ and τ^W^ ≪ τ^unif^, and thus *f*() = 0. This leads to the exchange energy only being determined by the UEG exchange enhancement factor *F*_x_.^1^ Hence, the interpolation function and inhomogeneity parameter allow the functional to switch between different density regimes.

The expression for  is taken from r^2^SCAN^36^ and is a regularized form of the expression used by Smeets et al.^10^ This regularization leads to a numerically more stable functional and mainly affects convergence in cases that are more difficult to converge due to a singularity in the nonregularized form of the inhomogeneity parameter. The regularization does not affect the results but only improves the numerical performance. We would like to emphasize that rMS-RPBEl-rVV10 is not a new DF. We have only introduced the regularization of the inhomogeneity parameter from r^2^SCAN^36^ into the MS-RPBEl-rVV10 DF designed by Smeets et al.^10^ Additionally, a different regularization of the iso-orbital indicator in the MS2 DF has been tried by Furness and Sun, where similar observations have been made.^44^

Two GGA DFs were also used to evaluate the performance of the rMS-RPBEl-rVV10 DF, namely, RPBE^23^ with Grimme’s D3 nonlocal correction^37^ and Bayesian error estimation functional with nonlocal van der Waals correlation^22,45^ (BEEF-vdW). These GGA DFs were only used for calculations on the Cu(111) slab. The core electrons were described by the projector-augmented wave method.^46,47^ A plane-wave kinetic energy cutoff of 600 eV was used for the plane-wave basis set, and the energy in the self-consistent field was converged to within 10^–7^ eV.

The Cu lattice constant is optimized using a Γ-centered 20 × 20 × 20 *k*-point mesh. The force on each atom is converged to within 0.005 eV/Å. The lattice constant computed with the rMS-RPBEl-rVV10 DF is 3.52 Å, which is the same value as the one reported by Smeets and Kroes^33^ and is in good agreement with the experimental value of 3.60 Å.^48^ For the BEEF-vdW and RPBE-D3 DFs, the lattice constants are 3.66 and 3.58 Å, respectively.

The Cu(211) and Cu(111) surfaces are both modeled as a 3 × 3 periodic 6-layer slab with a 16 Å vacuum region placed between periodically repeated slabs. During the calculations, the three upper layers and adsorbates are fully relaxed, while the lower layers remain fixed at equilibrium bulk positions. For both slabs, a Γ-centered 4 × 4 × 1 *k*-point mesh is used for sampling the Brillouin zone. The force on each atom is converged to within 0.01 eV/Å. First, the interlayer distance is optimized with these settings. For the Cu(111) slab, only the *z*-coordinate of the atoms of the top three layers is allowed to relax, while the *x*- and *y*-coordinates are fixed. In all other calculations, all coordinates are relaxed. The distance between the top two layers in the Cu(111) slab increases by 1.8% for the rMS-RPBEl-rVV10 functional, which is in good agreement with the value reported by Smeets and Kroes,^33^ i.e., an increase of 1.6%. For the BEEF-vdW and RPBE-D3 DFs, the interlayer distance decreased by 1.0% and increased by 1.1%, respectively. The distance between the top two 111 layers in the Cu(211) slab increased with 3.8% for the rMS-RPBEl-rVV10 DF.

TSs are obtained using the dimer method as implemented in the VASP TS tools^49−52^ package and are confirmed to be first-order saddle points by checking if only one imaginary frequency is found in the normal-mode analysis. In TS searches for dissociative chemisorption reactions, the surface was fixed to the optimized geometry as in these reactions the surface does not have enough time to rearrange.

The adsorption energy of species, *E*_ads_, is defined as
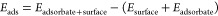
where *E*_adsorbate+surface_, *E*_surface_, and *E*_adsorbate_ are the total energies of the adsorbate on the slab, the clean Cu slab, and the gaseous adsorbate, respectively. The convergence of the calculation parameters is tested and is provided in Supporting Information S.1.

## 3 Results and Discussion

### 3.1 Comparison of DFs

The calculated Cu lattice constants of RPBE-D3, BEEF-vdW, and rMS-RPBEl-rVV10 are 3.575, 3.664, and 3.525 Å, respectively, while the experimental value is 3.597 Å.^48^ The inclusion of vdW corrections influences the prediction of the lattice constant, i.e., the lattice constant tends to decrease when including vdW corrections, which is beneficial for RPBE and unfavorable for the MS mGGA DFs,^10^ as they accurately predict the lattice constant without vdW corrections, while RPBE overestimates the lattice constant. Among the studied DFs, BEEF-vdW is found to overestimate the lattice constant, while RPBE-D3 and rMS-RPBEl-rVV10 demonstrate underestimation. Both the RPBE-D3 and the BEEF-vdW lattice constants are slightly closer to the experimental value than the value predicted by rMS-RPBEl-rVV10. Nevertheless, all exhibit reasonable predictions.

**Figure 2:**
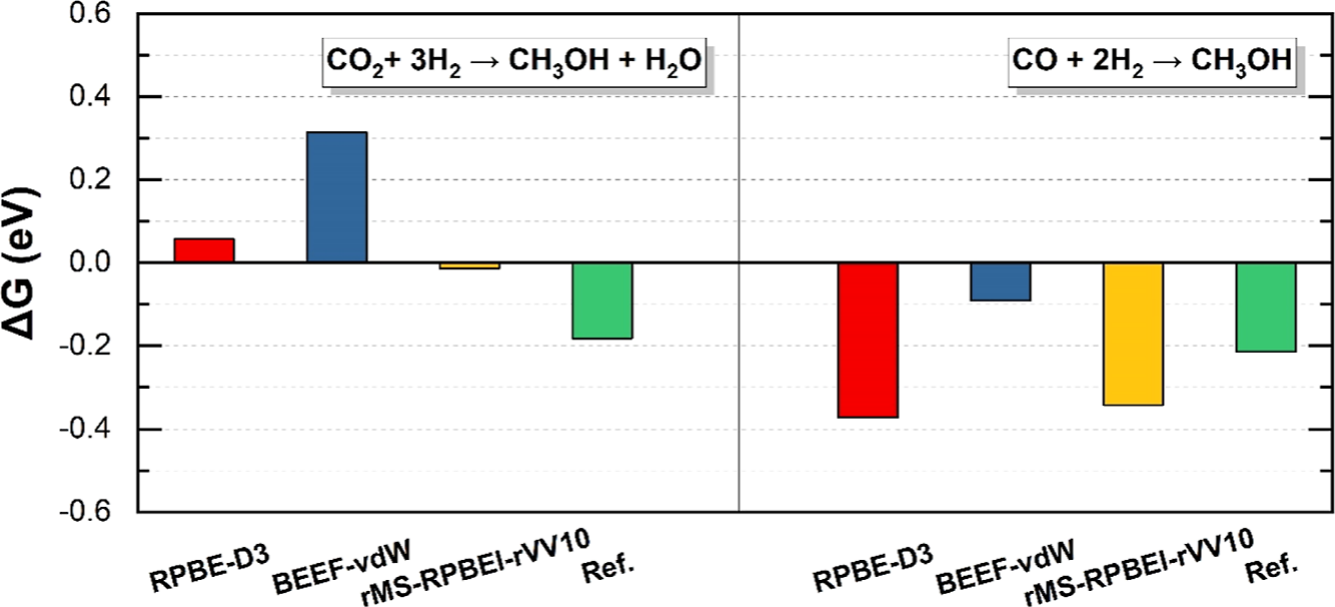
Comparison of Δ*G* in CO_2_ (left) and CO (right) hydrogenation calculated by RPBE-D3, BEEF-vdW, and rMS-RPBEl-rVV10 with the reference value.^3^ The thermal correction is at 500 K and the pressures of CO_2_, CO, H_2_, H_2_O, and CH_3_OH are 10, 10, 40, 1, and 1 bar, respectively.

In Figure 2, the change of the Gibbs free energy (Δ*G*) was calculated for the gas-phase reactions CO_2_ + 3H_2_ → CH_3_OH + H_2_O and CO + 2H_2_ → CH_3_OH, represented as Δ*G*_CO_2__ and Δ*G*_CO_, respectively, utilizing three DFs. Thermodynamic corrections (see S.2 in Supporting Information) were applied for typical operating conditions in the industrial hydrogenation of CO_2_ to methanol. These conditions include a temperature of 500 K and pressures of CO_2_, CO, H_2_, H_2_O, and CH_3_OH at 10, 10, 40, 1, and 1 bar, respectively. The obtained results indicate that all three DFs give reasonable predictions of Δ*G*_CO_, with BEEF-vdW overestimating Δ*G*_CO_ (i.e., not negative enough) with 0.12 eV, rMS-RPBEl-rVV10 underestimating Δ*G*_CO_ with 0.13 eV, and RPBE-D3 underestimating Δ*G*_CO_ with 0.16 eV. For Δ*G*_CO_2__, on the other hand, both BEEF-vdW and RPBE-D3 predict the reaction to be thermodynamically unfavorable, which can likely be ascribed to inaccuracies in addressing the O–C–O backbone with GGA DFs. Hence, corrections of gas-phase molecules are typically necessary for CO_2_-related studies based on GGA DFs.^21,53^ Of the used DFs, rMS-RPBEl-rVV10 offers the most accurate prediction of Δ*G*_CO_2__ by predicting an exothermic reaction. Furthermore, the difference between Δ*G*_CO_2__ and Δ*G*_CO_ is also best predicted by rMS-RPBEl-rVV10. In short, rMS-RPBEl-rVV10 does not only yield reasonable predictions for metal surfaces but also for gas-phase molecules, which is fundamentally not possible with GGA DFs.^4^

Now, we turn to the adsorption of important reaction intermediates on a Cu(111) surface by again comparing the aforementioned DFs. The most favorable adsorption sites, i.e., the sites with the lowest adsorption energy, along with their respective adsorption energies are presented in Table 1. All three DFs predict the same adsorption sites. Both H_2_ and CO_2_ demonstrate weak adsorption on the surface, exhibiting physisorption characteristics in line with previously reported findings.^62,63^ A more complete overview of adsorbed intermediates calculated with rMS-RPBEl-rVV10 on Cu(111) and Cu(211) can be found in S.3 in Supporting Information.

**Table 1:** Adsorption Energies, Dissociation Barriers, and Preferred Binding Sites of Relevant Species on Cu(111), Calculated with RPBE-D3, BEEF-vdW, and rMS-RPBEl-rVV10^a^

	RPBE-D3	BEEF-vdW	rMS-RPBEl-rVV10	site	ref value (eV)
Adsorption Energy (eV)					
CO	–0.84	–0.53	–0.87	fcc	–0.57^54^
H_2_O	–0.39	–0.19	–0.25	top	–0.40^55^
HCOOH	–0.53	–0.26	–0.38	top	–0.55^56^
CH_2_O	–0.33	–0.16	–0.25	hcp	–0.10^57^
CH_3_OH	–0.51	–0.26	–0.38	top	–0.60^58^
CO_2_	–0.26	–0.17	–0.12	phys	–0.25^59^
H_2_	–0.10	–0.05	–0.02	phys	–0.03^60^
Dissociation Barrier (eV)					
H_2_	0.47	0.94	0.57	bridge	0.63^25^
CO_2_	1.34	1.55	1.29	fcc	0.96^61^
Errors (eV)					
MSD	–0.08	0.15	0.02		
MAD	0.11	0.17	0.15		

a Phys indicates physisorbed species. The CO_2_ dissociation barrier is not taken into account for the calculation of the MAD and MSD.

In terms of the adsorption energy of CO, rMS-RPBEl-rVV10 and RPBE-D3 predict a considerably lower energy compared to that of the experiment (cf. ref. value in last column). The adsorption energies predicted by RPBE-D3, BEEF-vdW, and rMS-RPBEl-rVV10 are −0.84, −0.53, and −0.87 eV, respectively. An experimental value of −0.57 eV was reported by Hinch and Dubois using high-resolution electron-energy loss spectroscopy.^54^ Notably, the description of CO adsorption on transition metal surfaces, such as Cu(111), poses significant complications in DFT calculations, a dilemma referred to as the “CO adsorption puzzle”.^64,65^ Experimental evidence has suggested a preference for CO to adsorb at the top site on Cu(111).^66^ However, all three DFs employed in this study indicate that the favored adsorption site is the fcc site. This suggests that the reasonable agreement of the adsorption energy predicted by BEEF-vdW with the experiment is a consequence of cancellation of errors and not of its predictive capability. This phenomenon can be attributed to a synergistic effect of the SIE and errors driven by density approximations.^67^ A notable solution to this quandary was recently provided by Mishima et al.,^68^ who applied a long-range corrected hybrid DF, thereby mitigating the issue. In light of these findings, it becomes intriguing to explore the utilization of higher-level DFs, such as hybrid mGGA DFs, in order to achieve a more accurate description of reactions related to CO by minimizing the influence of the SIE. To the best of our knowledge, no hybrid GGA studies of a CO_2_ reaction network on a Cu surface are available in literature. Hence, there is no comparison with such a study in this work. Also, for the foreseeable future, such DFs are intractable, due to their computational cost, for computing an entire reaction network that includes explicitly the barrier heights.

For H_2_ and CO_2_, energy barriers for dissociative chemisorption were further calculated with the three DFs. For H_2_, rMS-RPBEl-rVV10 is 0.10 eV closer to the benchmark value of 0.63 eV^25,69^ than RPBE-D3. BEEF-vdW overestimates the barrier by 0.31 eV. It also fails in predicting the CO_2_ dissociation barrier, i.e., the BEEF-vdW value is the furthest away from the reference value. This can likely be ascribed to inaccuracies in addressing the O–C–O backbone in the BEEF-vdW functional. The value predicted by rMS-RPBEl-rVV10 is closest to the reference value. It has to be noted that the reference value for CO_2_ dissociation is the energy barrier for dissociation on a Cu(100) surface and only serves to give an indication of the barrier height as, to the best of our knowledge, there is no suitable reference value on Cu(111) in literature. Hence, we did not include the CO_2_ dissociation barrier energies in the calculation of the mean signed deviation (MSD) and the mean absolute deviation (MAD) in Table 1.

Dissociative adsorption of O_2_ onto the Cu(111) surface has been a topic of debate, mainly because O_2_ has triplet spin in its ground state.^70^ Helium atom scattering experiments^71^ have indicated an energy barrier for O_2_ dissociation on the Cu(111) surface, while DFT calculations using GGA-level DFs often predict it as a spontaneous process.^72^ The transition from the triplet state to the singlet state often introduces inaccuracies in the description of the electronic density with GGA-level DFs, leading to a rather poor description of the activated character.^73^ Notably, the dimer method for TS search proved ineffective for RPBE-D3 and BEEF-vdW DFs. Hence, we constructed 2D cuts of the 6D potential energy surface (PES) by varying the O_2_-surface distance and O–O bond length on the bridge site of Cu(111) with the three DFs. Figure S6 shows these elbow plots. From the elbow plots, it is clear that there is no barrier predicted by RPBE-D3 and BEEF-vdW. In contrast, the rMS-RPBEl-rVV10 elbow plot demonstrates a noticeable energy barrier between the physisorption well at higher distances from the surface and the dissociative chemisorption well at lower distances from the surface and higher O–O bond lengths. Although the absolute energy of the top of the barrier is slightly lower, i.e., −0.05 eV, than the reference energy of O_2_ and the surface far away from each other, it offers a qualitatively more accurate description compared to GGA-level DFs as they predict no barrier at all. As both RPBE-D3 and BEEF-vdW do not predict a barrier, O_2_ dissociation was not included in Table 1 and the calculation of the MSD and MAD. We also note that, in general, the MS DFs yield superior performance so far over GGA DFs for the prediction of barrier heights for systems that exhibit a large amount of charge transfer.^6,32^ Hence, rMS-RPBEl-rVV10 seems to outperform GGA DFs in predicting molecule–metal surface reaction barriers.

Based on the tabulated values, the MSD values for the RPBE-D3, BEEF-vdW, and rMS-RPBEl-rVV10 are determined to be −0.08, 0.15, and 0.02 eV, respectively. The MAD values corresponding to these DFs are calculated to be 0.11, 0.17, and 0.15 eV, respectively. In general, the GGA-DFs with vdW corrections, especially RPBE-D3, perform similar to rMS-RPBEl-rVV10 for adsorption energies but fail at predicting barriers for dissociation, aligning with the consensus that GGA DFs systematically get the barriers wrong.^6^ Correctly predicting these barriers is crucial for the development of a metal surface reaction network. Hence, we propose that for intricate reaction networks such as CO_2_, hydrogenation to CH_3_OH rMS-RPBEl-rVV10 demonstrates superior predictive capabilities with respect to GGA DFs. This mGGA-level DF with vdW correction likely attains a reasonable level of prediction by partially mitigating the SIE as well as by distinguishing the molecular and metallic regimes.

**Figure 3:**
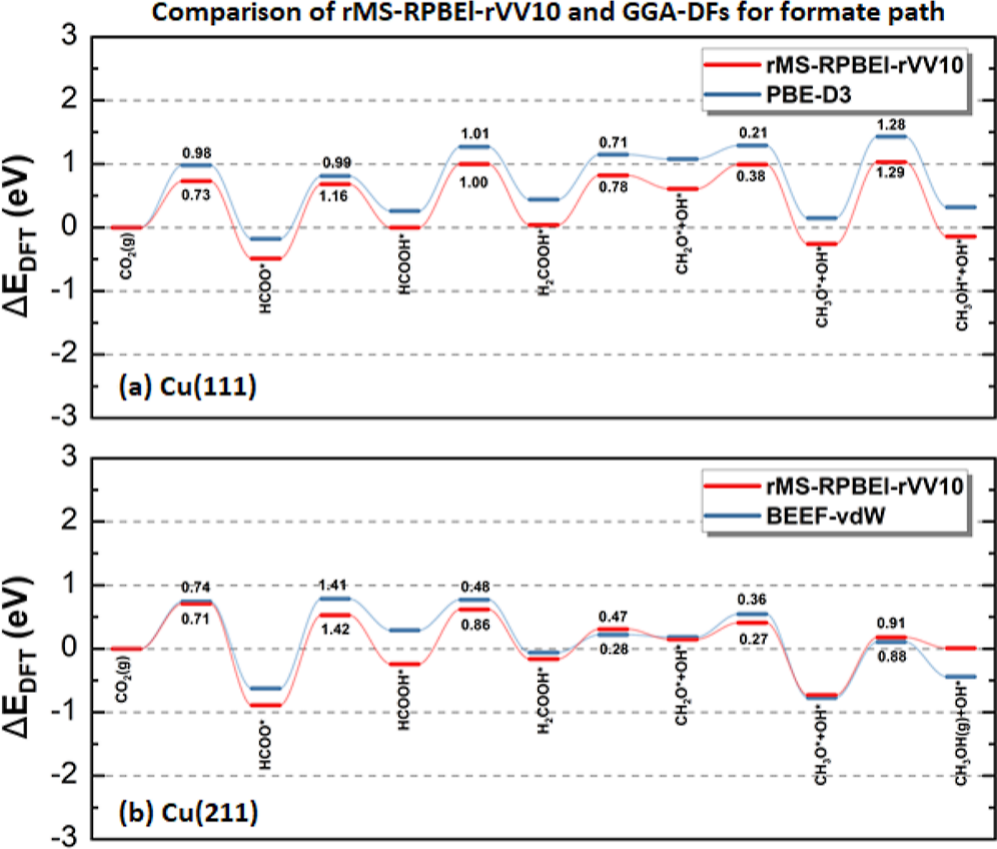
Potential energy diagram for the optimal formate pathway computed with (a) PBE-D3 (blue) and rMS-RPBEl-rVV10 (red) on Cu(111) and (b) BEEF-vdW (blue) and rMS-RPBEl-rVV10 (red) on Cu(211). To simplify, H* are not listed. Activation energy barriers are denoted in eV.

In order to further evaluate the performance of rMS-RPBEl-rVV10 for the CO_2_ reaction network, we make a comparison with state-of-the-art studies including van der Waals (vdW) corrections. For the formate pathway on Cu(111), we compare our rMS-RPBEl-rVV10 results with the results of Shi et al.,^74^ who employed the PBE-D3 DF (Figure 3a), since RPBE-D3 results are missing from literature. For the formate pathway on Cu(211), we compare our results to those of Studt et al.,^21^ who employed the BEEF-vdW DF (Figure 3b). All of the corrections, i.e., empirical energy corrections for gas-phase molecules that have an O–C–O bond, are removed for a clear comparison.

Both PBE-D3 and rMS-RPBEl-rVV10 predict that the process’s highest activation barrier on Cu(111) occurs during the hydrogenation of CH_3_O* to CH_3_OH*. It is important to note that the majority of the reaction barriers calculated using rMS-RPBEl-rVV10 tend to be marginally higher than those obtained with PBE-D3. As discussed earlier, this discrepancy may be ascribed to the difficulties GGA DFs have in predicting reaction barriers. However, an exception can be observed in the reaction step CO_2_(g) + H* → HCOO*, which might involve the difference of the description of gas-phase molecules by both DFs.

In contrast to Cu(111), on Cu(211), BEEF-vdW and rMS-RPBEl-rVV10 both predict HCOO* hydrogenation to have the highest barrier. Note that the results published by Studt et al. contain empirical corrections, which we removed here.^17^ Overall, rMS-RPBEl-rVV10 again predicts higher energy barriers than BEEF-vdW.

### 3.2 Analysis of CH_3_OH Formation Pathways

**Figure 4:**
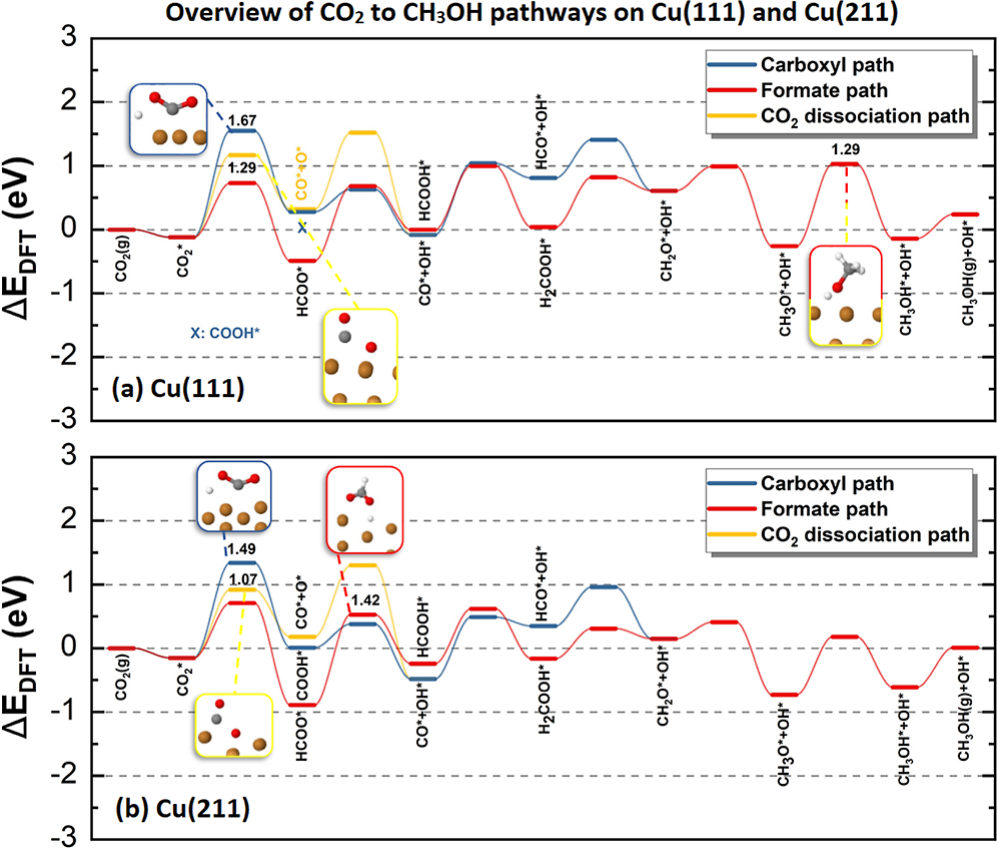
Potential energy diagram of possible CO_2_ hydrogenation pathways on (a) Cu(111) and (b) Cu(211) computed with rMS-RPBEl-rVV10. The carboxyl path is in blue, the formate path is in red, and the CO_2_ dissociation path is in yellow. To simplify, all H* are not listed. TS geometries of the rate-controlling step and their energy (in eV) for each pathway, as well as intermediate states, are depicted in the figure.

Finally, we compare the three different pathways on Cu(111) and Cu(211) computed with rMS-RPBEl-rVV10 in Figure 4. A more detailed discussion of all possible pathways can be found in S.6 in the Supporting Information. On Cu(111), the formate path proceeds through HCOOH* rather than H_2_COO* and the carboxyl path through CO* rather than COHOH*. The rate-controlling steps for each path are indicated in Figure 4a. Based on the rate-controlling step, the formate and CO_2_ dissociation pathways are equally favorable as the highest barriers have the same height for both paths and are lower than the highest barrier in the carboxyl path. The CO_2_ dissociation has two possible rate-controlling steps that have the same barrier, namely, CO_2_* dissociation and CH_3_O* hydrogenation; both TSs are depicted in the figure. As the rate-controlling barriers in the CO_2_ dissociation pathway and formate pathway are the same height, it is difficult to draw a conclusion about the most favorable pathways on Cu(111). These results underline the importance of the DF as the small differences due to the choice of DF between pathways can easily change their relative importance.

On Cu(211), the formate and the carboxyl path proceed through the same intermediates as on Cu(111). The rate-controlling step for the formate route is the hydrogenation of HCOO* to HCOOH*, which is different from the rate-controlling step on Cu(111), namely, CH_3_O* hydrogenation. The rate-controlling step in the carboxyl path is the formation of COOH*, the same as on Cu(111). For the CO_2_ dissociation pathway, CO_2_* dissociation is the rate-controlling step, again the same as on Cu(111). Based on the rate-controlling step, the CO_2_ dissociation pathway is the most favorable on Cu(211) as it has the lowest rate-controlling barrier.

In general, the intermediate states are more stable, relative to gaseous CO_2_, on Cu(211) than on Cu(111), and the reaction barriers are lower on Cu(211). This is illustrated in Figure S13 for the formate pathway. The mean difference in activation energy barriers for all elementary reactions in the three pathways leading to CH_3_OH formation, calculated as *E*_a_(111) – *E*_a_(211), is 0.13 eV. This indicates that, in general, the differences are small and that there are important exceptions like the hydrogenation of HCOO*, which has a higher barrier on Cu(211).

The experimental affirmation of a CO promotion effect, as indicated by our calculations that point to the importance of CO in the CO_2_ dissociation path on both facets, in the synthesis of methanol from a CO_2_/H_2_ mixture is well-accepted, yet the reason for this effect remains unclear. It has been suggested that this effect is due to CO promoting HCOO* hydrogenation.^75^ Other studies posit that the CO concentration in the gas phase could enhance morphological modifications on the catalyst particles that can potentially explain the observed promotion effect on catalytic activity.^76^ Both of the former mechanisms are, of course, not captured by our DFT calculations. It is worth noting that the highest energy barrier in the formate pathway on the Cu(211) surface occurs in the hydrogenation of HCOO* to HCOOH*. In contrast, for the formate path on the Cu(111) surface, the highest energy barrier occurs in the hydrogenation of CH_3_O* to CH_3_OH*. Experiments confirmed the presence of surface HCOO* during CO_2_ hydrogenation to methanol on several Cu-based catalysts.^77,78^ Furthermore, similar to HCOO*, infrared experiments revealed the presence of CH_3_O* on Cu-based catalysts.^54,77^ Edwards and Schrader^75^ determined through in situ infrared testing that the hydrogenation of CH_3_O* on Cu-based catalyst surfaces is the rate-controlling step for CH_3_OH production, similar to our results on Cu(111). The presence of these intermediates is something that would be expected on the basis of our calculations, i.e., the hydrogenation of CH_3_O* is a rate-controlling step on Cu(111) and HCOO* is the most stable intermediate, relative to gas-phase CO_2_, on Cu(211) and has the second highest barrier to be hydrogenated on Cu(211).

From the discussion above, we cannot draw any definite conclusion about which pathway is responsible for CO_2_ conversion to CH_3_OH on either Cu surface. For example, the CO_2_ dissociation pathway has the lowest rate-controlling barrier on Cu(211) but goes through HCO*, which is kinetically unstable, as it is likely to react back to H* and CO* because this reaction has a lower barrier than that of hydrogenation to CH_2_O*. Furthermore, several intermediates, like CH_2_O*, are only weakly adsorbed, and desorption of these intermediates could influence the mechanism. It is hard, if not impossible, to draw a conclusion about the pathways based on DFT data alone. We plan to construct a microkinetic model to analyze the reaction network in more detail as all these different aspects can be included in such a model. Nevertheless, our DFT calculations do provide valuable insights, and it is the first time, to the best of our knowledge, that all these pathways are investigated in one study. Some of the studies mentioned in this paper only study one pathway, and most of them do not include CO_2_ dissociation, which, according to our calculations, is the most favorable pathway on Cu(211). It is also the first time that this system is investigated with a mGGA DF, which should indeed yield more robust results than with GGA DFs. We emphasize that it is important that the employed DF is relatively robust with respect to the various pathways for the calculations to be able to make predictions.

## 4 Conclusions

In summary, this study investigates for the first time the performance of a mGGA-level DF in predicting reaction networks on a metal surface. We compare the new rMS-RPBEl-rVV10 mGGA DF with the representative (R)PBE-D3 and BEEF-vdW GGA DFs using the catalytically important CO_2_ hydrogenation process on the Cu(111) and Cu(211) surfaces. The calculation of Δ*G* for CO and CO_2_ hydrogenation in the gas phase shows that rMS-RPBEl-rVV10 provides the closest prediction to the reference value for Δ*G*_CO_2__ without requiring empirical corrections for a qualitatively correct description, which has been typically a necessity with GGA DFs so far. Furthermore, the adsorption and activation energies of various intermediates and reactions on Cu(111) are investigated. All three DFs predict similar adsorption and TS geometries but show discrepancies in the adsorption and activation energies. While RPBE-D3 has the lowest MAD (0.11 eV), rMS-RPBEl-rVV10 also yields low MSD and MAD (0.02 and 0.15 eV, respectively). rMS-RPBEl-rVV10 performs similarly to the GGA DFs for adsorption but performs significantly better in predicting dissociation barriers, which are often critical in reaction networks. Together with the improved description of gas-phase molecules compared to GGA DFs and the similar performance for the prediction of adsorption energies and lattice constants, this indicates an outstanding performance of the mGGA DF for reaction networks. By comparing to other state-of-the-art studies, particularly for the formate pathways on the Cu(111) and Cu(211) surfaces, we find that rMS-RPBEl-rVV10 generally predicts higher energy barriers for surface reactions than GGA-level DFs and shows promise for more accurate predictions of CO_2_ hydrogenation processes. Also, by comparing different reaction pathways, we show that it is important for a DF to yield robust performance across a reaction network as the relative importance of pathways can be dependent on the choice of level of theory of the DF. This is in contrast to choosing a different DF at the same level of theory as the barriers will be shifted in a similar way in that case, not altering the relative importance. For example, our rMS-RPBEl-rVV10 results show that CO_2_ dissociation is one of the most favorable pathways on Cu(211), while typical GGA DF-level studies in literature dismiss this pathway. However, definitive conclusions regarding the CO_2_ conversion pathway to CH_3_OH on Cu(211) or Cu(111) surfaces require further analysis using a microkinetic model. Unfortunately, rMS-RPBEl-rVV10 does not resolve the “CO adsorption puzzle”, illustrating the need for the development of hybrid mGGA DFs that solve the SIE. Likewise, development of a more advanced vdW DF that takes advantage of the KED to supplement a (hybrid) mGGA DF should yield a considerable improvement over the employed rVV10 nonlocal correlation DF. In short, the use of a mGGA DF yields superior performance over GGA DFs for catalytically relevant reaction networks on metal surfaces. Considering the reasonable increase of computational cost, up to a factor of three, we recommend that future development of state-of-the-art reaction networks makes use of mGGA DFs instead of GGA DFs as mGGA DFs significantly improve the description of the gas phase and reaction barriers.

## Supporting Information Available

Convergence of computational parameters for HCOOH adsorption energy; formulas for the calculation of Gibbs free energy; adsorption energy and adsorption sites for all intermediates on Cu(111) and Cu(211) calculated with rMS-RPBEl-rVV10; list of (adsorbed) species and corresponding energies, entropies, zero-point energy, and thermal corrections calculated with rMS-RPBEl-rVV10, BEEF-vdW, and RPBE-D3; 6D PES of O_2_ dissociation on the Cu(111) surface; and detailed analysis of formate, carboxyl, and CO_2_ dissociation pathways on Cu(111) and Cu(211) (PDF)
