# Supplementary material for: Improving Molecule–Metal Surface Reaction Networks Using the Meta-Generalized Gradient Approximation: CO2 Hydrogenation
Source: J Phys Chem C Nanomater Interfaces. 2024 May 17;128(21):8611–20. doi: 10.1021/acs.jpcc.4c01110 (PMC11145648; doi:10.1021/acs.jpcc.4c01110)
Supplement: Supplementary file 1 — jp4c01110_si_001.pdf [file jp4c01110_si_001.pdf]

Supporting Information: Improving Molecule-Metal Surface Reaction Networks Using the Meta-Generalized Gradient Approximation: CO_2 Hydrogenation

Yuxiang Cai[a, b, +], Roel Michiels[a, +], Federica De Luca[a,c], Erik Neyts[a], Xin Tu[b], Annemie Bogaerts[a], Nick Gerrits[a, d, *]

[a] Research group PLASMANT, Department of Chemistry, University of Antwerp, Universiteitsplein 1, BE-2610 Wilrijk-Antwerp, Belgium

[b] Department of Electrical Engineering and Electronics, University of Liverpool, Liverpool L69 3GJ, United Kingdom

[c] Department of ChiBioFarAM (Industrial Chemistry), University of Messina, ERIC aisbl and INSTM/CASPE, V.le F. Stagno d'Alcontres 31, Messina 98166, Italy

[d] Leiden Institute of Chemistry, Gorlaeus Laboratories, Leiden University, P.O. Box 9502, 2300 RA Leiden, The Netherlands

[+] Shared first authors

* Corresponding author: n.gerrits@lic.leidenuniv.nl

S.1 Convergence of HCOOH adsorption energy

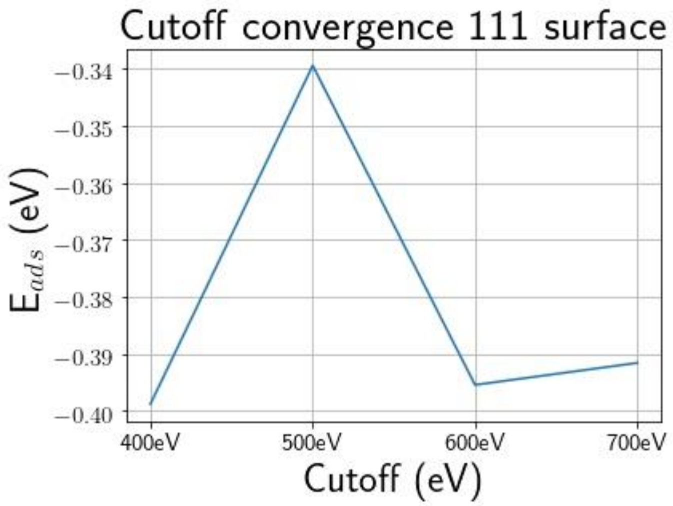

Figure S1: Convergence of the adsorption energy of HCOOH on Cu(111) as a function of the cutoff for rMS-RPBEl-rVV10.

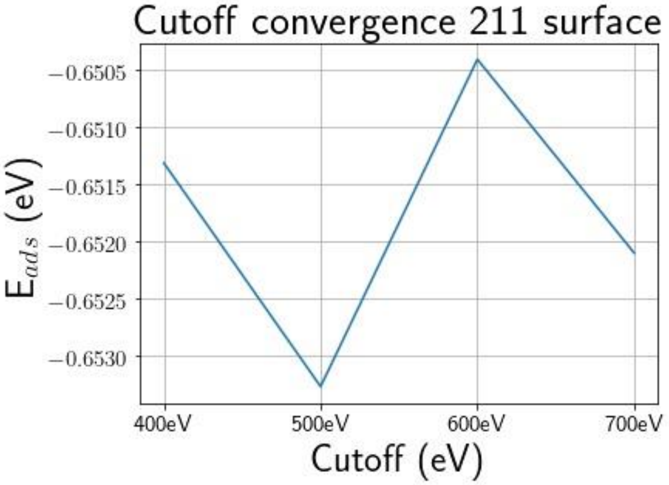

Figure S2: Convergence of the adsorption energy of HCOOH on Cu(211) as a function of the cutoff for rMS-RPBEl-rVV10.

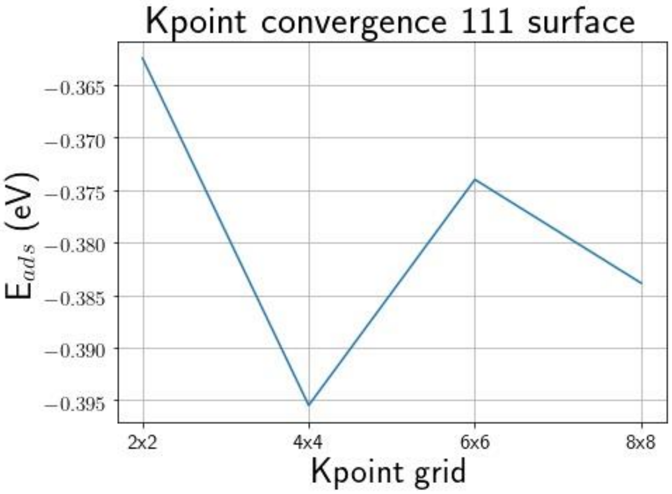

Figure S3: Convergence of the adsorption energy of HCOOH on Cu(111) as a function of the k-point grid for rMS-RPBEl-rVV10.

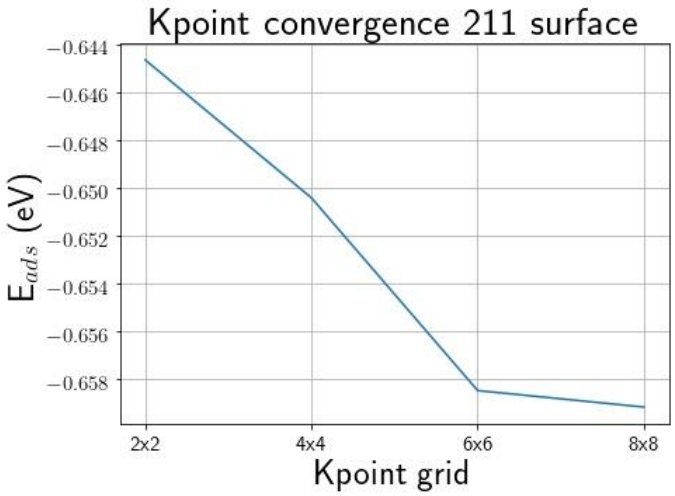

Figure S4: Convergence of the adsorption energy of HCOOH on Cu(211) as a function of the k-point grid for rMS-RPBEl-rVV10.

S.2 Formulas for zero-point energy, entropy and thermal corrections

S.2.1 List of symbols

N: number of atoms
h: Planck's constant
c: speed of light
$\tilde{\nu}_i$: wavenumber
k_B: Boltzmann constant
P: pressure
T: temperature
m: mass of the species
ε_i: energy of the vibrational mode
Θ: rotational temperature
I: moment of inertia
σ: symmetry number
$n_{\text{unpaired e-}}$: number of unpaired electrons
E_{ZPE}: zero point energy
S_{trans}: translational entropy at standard pressure
S_{vib}: vibrational entropy
S_{rot}: rotational entropy
S: total entropy
U_{trans}: translational energy
U_{vib}: vibrational energy
U_{rot}: rotational energy
H: enthalpy
C_p: isobaric heath capacity
G: Gibbs free energy

S.2.2 List of formulas for Gibbs free energy calculation

The wavenumber of imaginary modes and modes with a wavenumber lower than 50 cm^{-1} is taken to be 50 cm^{-1} in all calculations to avoid that $e^{\frac{\varepsilon_i}{k_\mathrm{B}T}} - 1$ approaches 0, which would cause U_vib and S_vib to blow up. 3N-6 becomes 3N-5 for linear molecules in the formulas below.

$$E_\mathrm{ZPE} = \sum_{i}^{3N-6} \frac{hc\tilde{v}_i}{2}$$

$$S_\mathrm{trans} = k_\mathrm{B}\left(\ln\left(\frac{k_\mathrm{B}T}{\mathrm{P}}\left(\frac{2\pi m k_\mathrm{B}T}{h^2}\right)^{3/2}\right) + \frac{5}{2}\right)$$

$$S_\mathrm{vib} = k_\mathrm{B}\sum_{i}^{3N-6}\left(\frac{\varepsilon_i}{k_BT\left(e^{\frac{\varepsilon_i}{k_\mathrm{B}T}} - 1\right)} - \ln\left(1 - e^{-\frac{\varepsilon_i}{k_\mathrm{B}T}}\right)\right)$$

$$\Theta = \frac{h^2}{8\pi^2 I}$$

$$S_\mathrm{rot} = k_\mathrm{B}\left(\ln\left(\frac{k_\mathrm{B}T}{\sigma\Theta}\right) + 1\right) \text{ (for linear molecule)}$$

$$S_\mathrm{rot} = k_\mathrm{B}\left(\ln\left(\frac{1}{\sigma}\left(\frac{\pi(k_\mathrm{B}T)^3}{\Theta_\mathrm{A}\Theta_\mathrm{B}\Theta_\mathrm{C}}\right)^{1/2}\right) + \frac{3}{2}\right) \text{ (for non-linear molecule)}$$

$$S(T) = S_\mathrm{trans}(T) + S_\mathrm{vib}(T) + S_\mathrm{rot}(T)$$

$$U_\mathrm{trans} = \frac{3}{2}k_\mathrm{B}T$$

$$U_{\text{vib}} = \sum_{i}^{3N-6} \frac{\varepsilon_i}{e^{\frac{\varepsilon_i}{k_{\text{B}}T}} - 1}$$

$$U_{\text{rot}} = k_{\text{B}}T \text{ (for linear molecule)}$$

$$U_{\text{rot}} = \frac{3}{2}k_{\text{B}}T \text{ (for non-linear molecule)}$$

$$\int_0^T C_p(T)dT = U_{\text{trans}}(T) + U_{\text{vib}}(T) + U_{\text{rot}}(T) + k_{\text{B}}T$$

$$H(T) = E + E_{\text{ZPE}} + \int_0^T C_p(T)dT$$

$$G(T) = H(T) - TS(T)$$

S.3 Adsorption energy and site of the intermediates

Table S1: Preferred binding sites and adsorption energies (E_{ads}) of the species involved in hydrogenation of CO_2 on Cu(211) and Cu(211) calculated with rMS-RPBEl-rVV10. Site H, Hs, s, Bs, Ts, fcc, hcp and Phy stand for 3-fold hollow, hollow step, step, bridge step, top step, face-centred cubic, hexagonal close-packed and physisorption. These sites are indicated on Figure S5 on the Cu(111) and Cu(211) surface.

	Cu(211)			Cu(111)		
Species	E_{ads} (eV)	site	*d*(Cu-A) (Å)	E_{ads} (eV)	site	*d*(Cu-A) (Å)
H*	-2.58	H (hcp)	Cu-H 1.72, 1.72, 1.82	-2.56	fcc	Cu-H 1.75, 1.75, 1.75
O*	-4.19	H (hcp)	Cu-O 1.88, 1.88, 1.94	-4.14	fcc	Cu-O 1.90, 1.90, 1.90
OH*	-2.98	Bs	Cu-O 1.95, 1.95	-2.66	fcc	Cu-O 2.76, 2.77, 2.77
HCO*	-1.60	Bs	Cu-C 2.07, 2.09	-1.43	C @bridge<br>O @ top	Cu-C 2.09, 2.08<br>Cu-O 2.10
CO_2*	-0.15	Phy	C-Surf 3.06	-0.12	Phy	C-Surf 3.10

H_2*	-0.06	Phy (Ts)	H-Surf 2.46<br>H-Cu 1.83	-0.02	Phy	H-Surf 3.17
H_2O*	-0.45	Ts	Cu-O 2.19	-0.25	top	Cu-O 2.33
CO*	-0.96	Bs	Cu-C 1.99, 1.98	-0.87	fcc	Cu-C 2.05, 2.05, 2.05
HCOOH*	-0.65	Ts	Cu-O 2.06	-0.38	top	Cu-O 2.15
CH_2O*	-0.45	Ts	Cu-O 2.09	-0.25	C @ top<br>O @ bridge	Cu-C 2.06<br>Cu-O 2.07, 2.07
CH_3OH*	-0.62	Ts	Cu-O 2.16	-0.38	top	Cu-O 2.23
HCOO*	/	O @ Ts<br>O @ Ts	Cu-O 1.95<br>Cu-O 1.95	/	O @ top<br>O @ top	Cu-O 2.00<br>Cu-O 2.00
H_2COO*	/	O @ Bs<br>O @ H	Cu-O 1.94, 1.97<br>Cu-O 2.08, 2.35, 1.97	/	O @ bridge<br>O@ bridge	Cu-O 2.03, 1.99<br>Cu-O 2.03, 1.98
H_2COOH*	/	O @ Bs<br>O @ top	Cu-O 1.96, 1.97<br>Cu-O 2.32	/	O @ bridge<br>O@ top	Cu-O 2.02, 2.02<br>Cu-O 2.30
COOH*	/	C @ Ts<br>O @ Ts	Cu-C 1.94<br>Cu-O 2.02	/	C @ top<br>O @ bridge	Cu-C 1.96<br>Cu-O 2.09
COHOH*	/	Ts	Cu-C 1.92	/	top	Cu-C 1.95
COH*	/	s	Cu-C 2.00, 2.01, 2.07, 2.05	/	fcc	Cu-C 1.95, 1.95, 1.92
HCOH*	/	s	Cu-C 2.07, 2.02	/	bridge	Cu-C 2.03, 2.03
H_2COH*	/	C @ Ts<br>O @ Ts	Cu-C 1.98<br>Cu-O 2.11	/	C @ top<br>O @ top	Cu-C 2.01<br>Cu-O 2.22

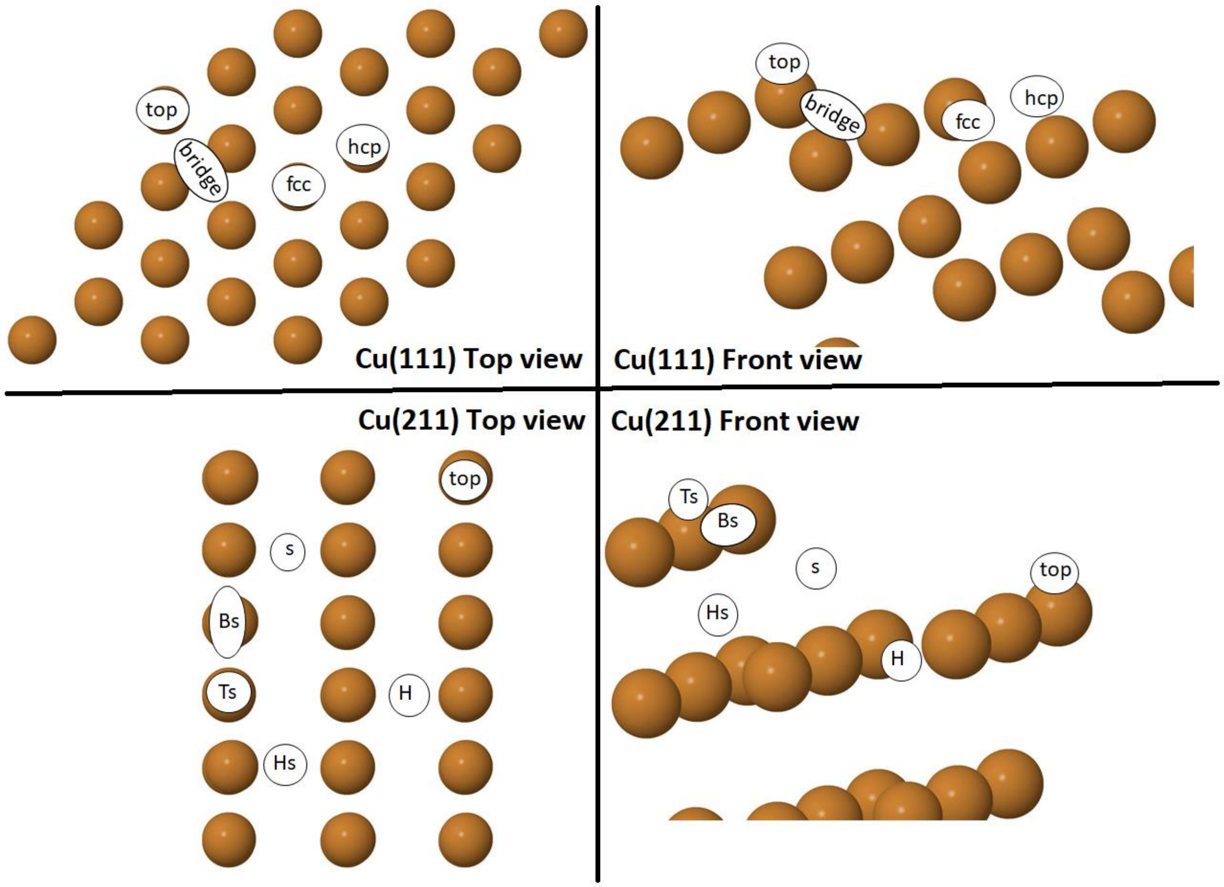

Figure S5: Sites on Cu(111) (top) and on Cu(211) (bottom). Sites are abbreviated as hcp-hexagonal close-packed, fcc-face-centred cubic, Bs-bridge step, Hs-hollow step, s-step, Ts-top step, H-3-fold hollow.

Table S1 provides a comprehensive overview of the adsorption configurations and adsorption energies of all reaction intermediates involved in the reaction network. The sites indicated in the table are depicted in Figure S5.

CO_2 does not show a specific adsorption morphology on the Cu(211) or Cu(111) surface. Both in the gaseous phase and physisorbed, CO_2 molecules maintain their linear structure, with C-O bond lengths of 1.18 Å, similar to the experimental measurement of 1.16 Å[1], which further strengthens the argument for physisorption of CO_2.

To the best of our knowledge, no experimental studies exist on H_2O adsorption on the Cu(211) surface. However, Brosseau et al.[2] studied H_2O adsorption on defect-rich Cu(100) surfaces, reporting an adsorption energy of approximately -0.52 eV, which is similar to the adsorption energy we find on the stepped Cu(211) surface.

CO adsorbs to the bridge site on the step, displaying an adsorption energy of -0.96 eV. Radnik et al.[3], employing electron energy loss spectroscopy techniques, studied the adsorption position and orientation of CO molecules on the Cu(211) surface, demonstrating that CO tends to adsorb on the top or bridge sites of the Cu(211) step surface, presenting an adsorption energy of -0.605 ± 0.015 eV.

Methanol synthesis might also proceed through the hydrogenation of intermediates like COHOH* and its derivatives. Schreiner et al.[4] identified three possible isomeric structures of COHOH, namely *t,t*-COHOH, and *t,c*-COHOH, through infrared spectroscopy and high-level ab initio coupled cluster theory calculations. Surface adsorption, however, was not included in this study. We find the most stable COHOH* to be the *t,t*-COHOH* configuration, which binds to the top site of the step on Cu(211).

S.4 List of species, energy, zero-point energy (ZPE) and thermal corrections

Table S2: List of adsorbates on Cu(211) and Cu(111) surface with corresponding total energies calculated with rMS-RPBEl-rVV10.

Adsorbate	*E* (eV)	
	Cu(211)	Cu(111)
Clean slab	371.43	370.85
H*	367.65	367.10
O*	364.95	364.42
OH*	360.51	360.26
H_2O*	356.94	356.56
CO*	355.44	354.95
CO_2*	348.63	348.08
HCOO*	344.11	343.96
HCOOH*	341.00	340.70
H_2COO*	341.22	340.89
H_2COOH*	337.31	336.99
COOH*	345.02	344.73
COHOH*	341.61	341.32
COH*	352.89	352.40
HCO*	352.50	352.09
HCOH*	349.13	348.57
CH_2OH*	344.62	344.45
CH_2O*	348.53	348.15
CH_3O*	343.88	343.52
CH_3OH*	340.22	339.89

Table S3: List of transition states on Cu(211) and Cu(111) surface with corresponding total transition state energies calculated with rMS-RPBEl-rVV10.

Reaction - TS	*E* (eV)	
	Cu(211)	Cu(111)
CO_2* + H* → HCOO* + *	345.72	345.18
CO_2(g) + H* → HCOO*	345.72	345.18
CO_2* + H* → COOH* + *	346.34	346.00
CO_2(g) + H* → COOH*	346.34	346.00
CO_2* + * → CO* + O*	349.58	349.25
CO_2(g) + 2* → CO* + O*	349.37	349.70
HCOO* + H* → HCOOH* + *	341.76	341.37

$HCOO^* + H^* \rightarrow H_2COO^* + *$	342.09	341.62
$HCOOH^* + H^* \rightarrow H_2COOH^* + *$	338.09	337.95
$H_2COO^* + H^* \rightarrow H_2COOH^* + *$	338.62	338.20
$H_2COOH^* + * \rightarrow CH_2O^* + OH^*$	337.78	337.77
$CH_2O^* + H^* \rightarrow CH_3O^* + *$	345.02	344.77
$CH_3O^* + H^* \rightarrow CH_3OH^* + *$	341.02	341.06
$CH_3O^* + H^* \rightarrow CH_3OH(g) + 2^*$	341.02	341.06
$COOH^* + * \rightarrow CO^* + OH^*$	345.39	345.08
$CO^* + H^* \rightarrow HCO^* + *$	352.64	352.33
$HCO^* + H^* \rightarrow CH_2O^* + *$	349.34	348.94
$COOH^* + H^* \rightarrow COHOH^* + *$	342.29	342.04
$COHOH^* + * \rightarrow COH^* + OH^*$	343.27	342.82
$COH^* + H^* \rightarrow HCOH^* + *$	350.32	349.38
$HCOH^* + H^* \rightarrow CH_2OH^* + *$	345.77	345.30
$CH_2OH^* + H^* \rightarrow CH_3OH^* + *$	341.67	341.35
$O^* + H^* \rightarrow OH^* + *$	362.29	361.87
$OH^* + OH^* \rightarrow H_2O^* + O^*$	350.95	350.18
$OH^* + H^* \rightarrow H_2O^* + *$	358.25	357.82
$H_2(g) + 2^* \rightarrow 2H^*$	364.39	364.92

Table S4: List of gas phase species with energies, zero-point energy, entropy and integrated heath capacity at 500 K calculated with rMS-RPBEl-rVV10.

Species	E (eV)	ZPE (eV)	S (eV/K)	$\int C_p dT$ (eV)
$H_2(g)$	-7.00	0.27	0.00120	0.15080
$CO_2(g)$	-22.65	0.31	0.00225	0.18532
$H_2O(g)$	-14.04	0.57	0.00214	0.17421
$CO(g)$	-15.03	0.13	0.00201	0.15136
$CH_3OH(g)$	-30.58	1.36	0.00291	0.24291
$HCOOH(g)$	-29.78			
$CH_2O(g)$	-22.45			
$O_2(g)$	-9.60			

Table S5: List of gas phase species with energies, zero-point energy, entropy and integrated heath capacity at 500 K calculated with BEEF-vdW.

Species	E (eV)	ZPE (eV)	S (eV/K)	$\int C_p dT$ (eV)
$H_2(g)$	-7.17	0.28	0.00119	0.15080
$CO_2(g)$	-18.41	0.31	0.00224	0.18539
$H_2O(g)$	-12.84	0.58	0.00215	0.17424
$CO(g)$	-12.09	0.13	0.00201	0.15139
$CH_3OH(g)$	-27.81	1.37	0.00276	0.23069
$HCOOH(g)$	-25.54			
$CH_2O(g)$	-19.60			
$O_2(g)$	-6.61			

Table S6: List of gas phase species with energies, zero-point energy, entropy and integrated heath capacity at 500 K calculated with RPBE-D3.

Species	*E* (eV)	ZPE (eV)	*S* (eV/K)	$\int C_p dT$ (eV)
H_2(g)	-6.98	0.27	0.00120	0.15081
CO_2(g)	-22.27	0.30	0.00225	0.18562
H_2O(g)	-14.16	0.57	0.00214	0.17437
CO(g)	-14.42	0.13	0.00201	0.15142
CH_3OH(g)	-30.04	1.35	0.00277	0.23172
HCOOH(g)	-29.31			
CH_2O(g)	-21.88			
O_2(g)	-9.57			

Table S7: List of adsorbates and transition states at Cu(111) surface with corresponding total energies calculated with BEEF-vdW and RPBE-D3.

Species	*E* (eV)	
	BEEF-vdW	RPBE-D3
Adsorbates		
Clean slab	-26.00	-199.20
CH_2O*	-45.75	-221.41
CH_3OH*	-54.07	-229.76
CO*	-38.62	-214.47
CO_2*	-44.58	-221.73
HCOOH*	-51.80	-229.05
H_2O*	-39.02	-213.75
H_2*	-33.22	-206.29
Reaction - TS		
CO_2(g) + 2* → CO* + O*	-42.86	-220.13
H_2(g) + 2* → H* + H*	-32.23	-205.72

S.5 2D (Z, r) cuts of the 6D potential energy surface (PES) of O_2 dissociation on Cu(111) surface.

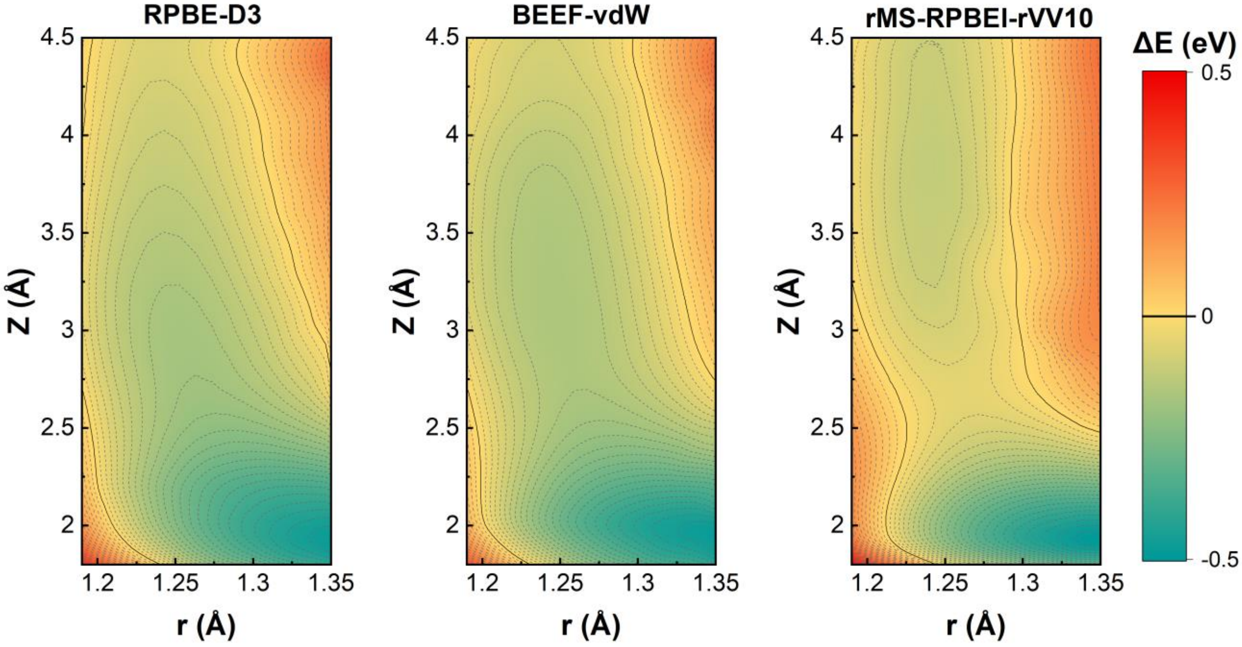

Figure S6. 2D (*Z, r*) cuts of the PES for O2/Cu(111) of O_2 dissociation on Cu(111) bridge site calculated with RPBE-D3, BEEF-vdW and rMS-RPBEl-rVV10, respectively. O_2 molecule is parallel to the surface. *Z* is the distance between O_2 molecule and the surface, *r* is the bond length between two O atoms. The solid lines represent the 0 eV reference value corresponding to O_2 and the surface in equilibrium and far away from each other. The energy difference between consecutive equipotential lines is 0.02 eV.

S.6 Detailed analysis of formate, carboxyl and CO_2 dissociation pathway on Cu surface.

S.6.1 Formate pathway

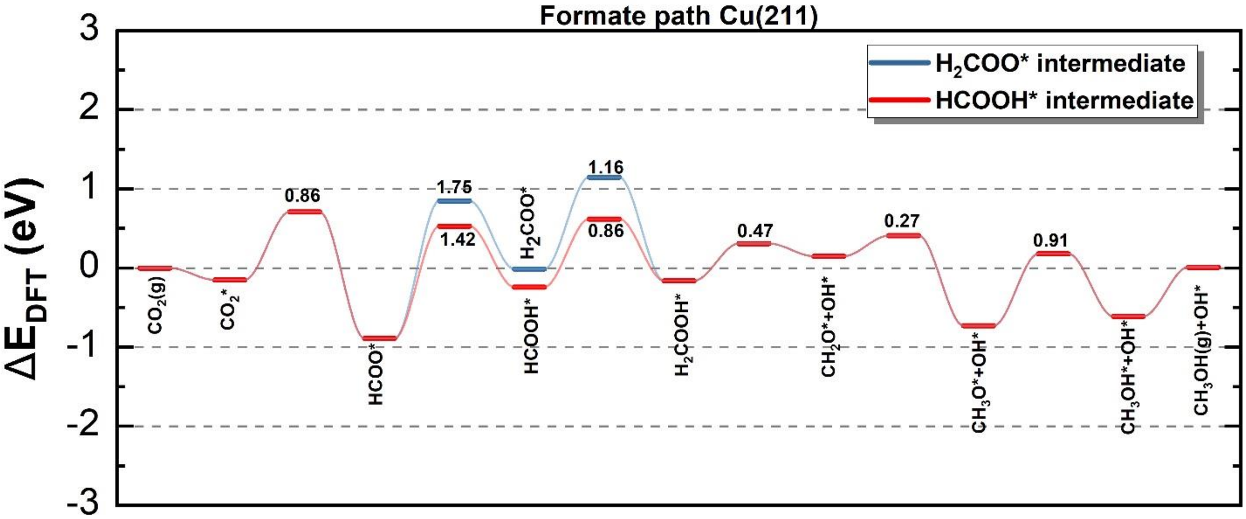

Figure S7: Potential energy diagram of both variants of the formate pathway on Cu(211), the path through H_2COO^* is indicated in blue, the path via HCOOH* in red. Activation energy barriers are in eV and intermediate states are depicted in the figure.

The potential energy diagram illustrating both variants of the formate pathway discussed in the introduction of the main paper is presented in Figure S7 for Cu(211). One pathway proceeds through the formation of H_2COO^*, indicated in blue, while the other goes through HCOOH*, indicated in red. The reaction energies of the hydrogenation of HCOO* to H_2COO^* and HCOOH* on Cu(211) are comparable, both reactions being endothermic and have reaction energies of 0.88 eV and 0.66 eV, respectively. However, the formation of H_2COO^* has an energy barrier that is 0.33 eV higher than the barrier for HCOOH* formation. This suggests a preference for the reaction to proceed via the formation of an O-H bond rather than a C-H bond. Furthermore, the subsequent HCOOH* hydrogenation to H_2COOH^* has a significantly lower energy barrier, i.e., 0.30 eV lower, than H_2COO^* hydrogenation. Therefore, on the Cu(211) surface, the pathway involving HCOOH* as an intermediate is more probable. The pathway can be summarized as CO_2^* → HCOO* → HCOOH* → H_2COOH^* → CH_2O^* → CH_3O^* → CH_3OH^*. The rate-controlling step is the hydrogenation of HCOO* to HCOOH* with an activation energy barrier of 1.42 eV.

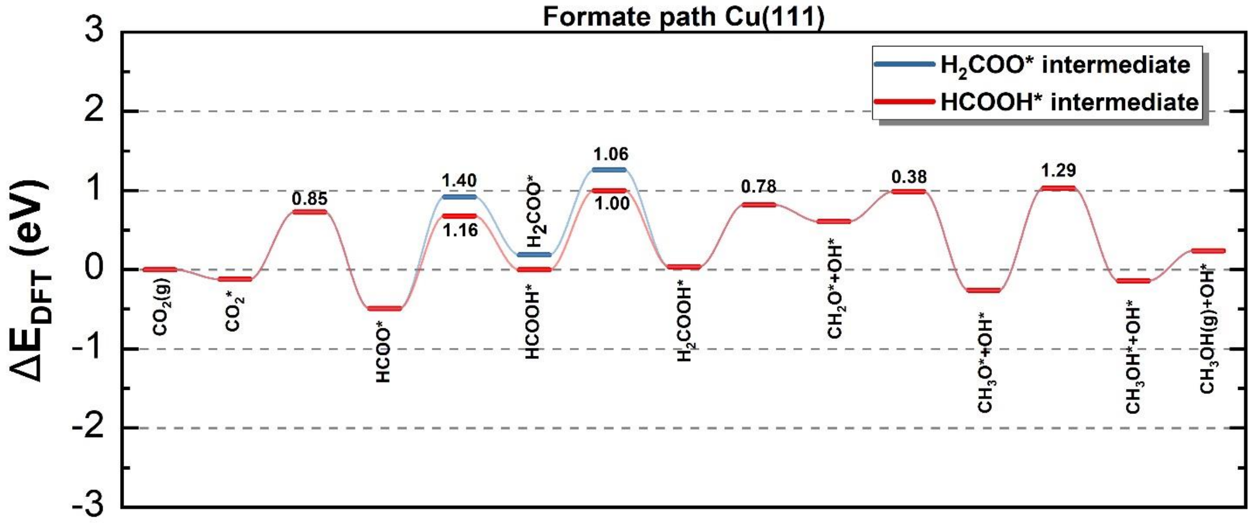

Figure S8: Potential energy diagram of both variants of the formate pathway on Cu(111), the path through H_2COO^* is indicated in blue, the path via HCOOH* in red. Activation energy barriers are in eV and intermediate states are depicted in the figure.

Both variants of the formate pathway on Cu(111) are depicted in Figure S8. From this figure, it is clear that the comparison between both variants of the formate pathway for Cu(111) is the same as for Cu(211). The most favourable formate pathway is the same for both facets, the rate-controlling step on Cu(111) is CH_3O^* hydrogenation. The barriers for the formate pathway through HCOOH* on Cu(111) are higher or similar to the barriers on Cu(211), except for HCOO* hydrogenation, which is 0.26 eV lower on Cu(111). The barriers for the pathway through H_2COO^* are lower on Cu(111).

S.6.2 Carboxyl pathway

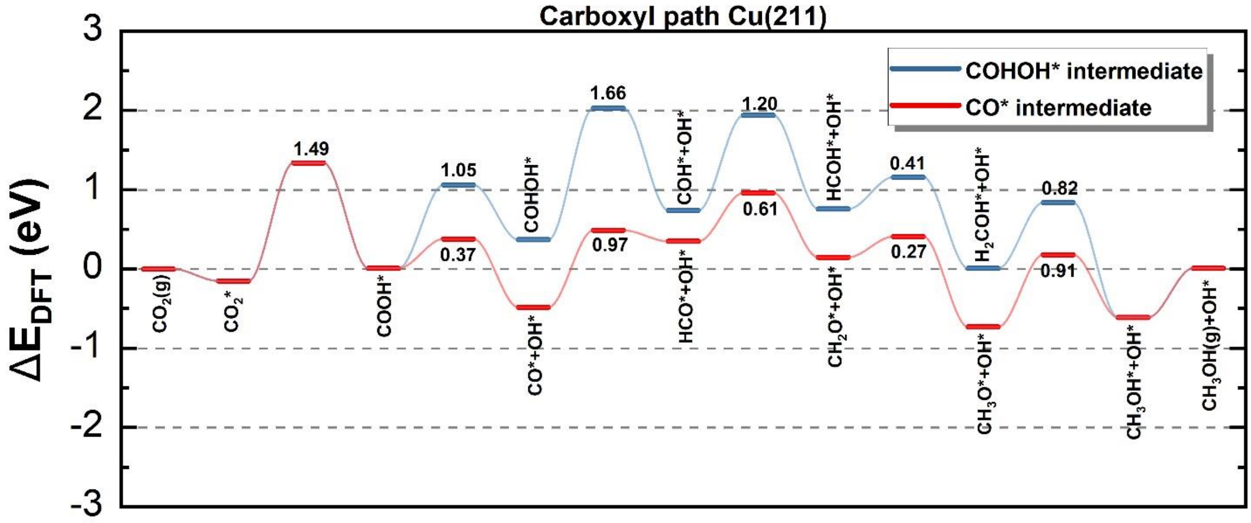

Figure S9: Potential energy diagram of both variants of the carboxyl pathway on Cu(211), the path through COHOH* is indicated in blue, the path via CO* in red. Activation energy barriers are in eV and intermediate states are depicted in the figure.

The potential energy diagram illustrating both variants of the carboxyl pathway discussed in the introduction is presented in Figure S9 for Cu(211). The COOH* intermediate can decompose into CO* and OH*, indicated in red, or react to COHOH*, indicated in blue. The energy barrier of the former reaction is 0.68 eV lower, indicating that the formation of CO* is kinetically favourable. Furthermore, this reaction is exothermic, while the reaction towards COHOH* is endothermic. Lastly, the subsequent dissociation of COHOH* has a barrier of 1.66 eV, which is considerably higher, i.e., at least 0.69 eV, than any barrier in the path through the CO* intermediate. Thus, upon comparing the different elementary reactions, we can conclude that the most favourable carboxyl pathway for methanol synthesis is CO_2* → COOH* → CO* → HCO* → CH_2O* → CH_3O* → CH_3OH*. The rate-controlling step for this pathway on Cu(211) is CO_2* hydrogenation to COOH* with an activation barrier of 1.49 eV.

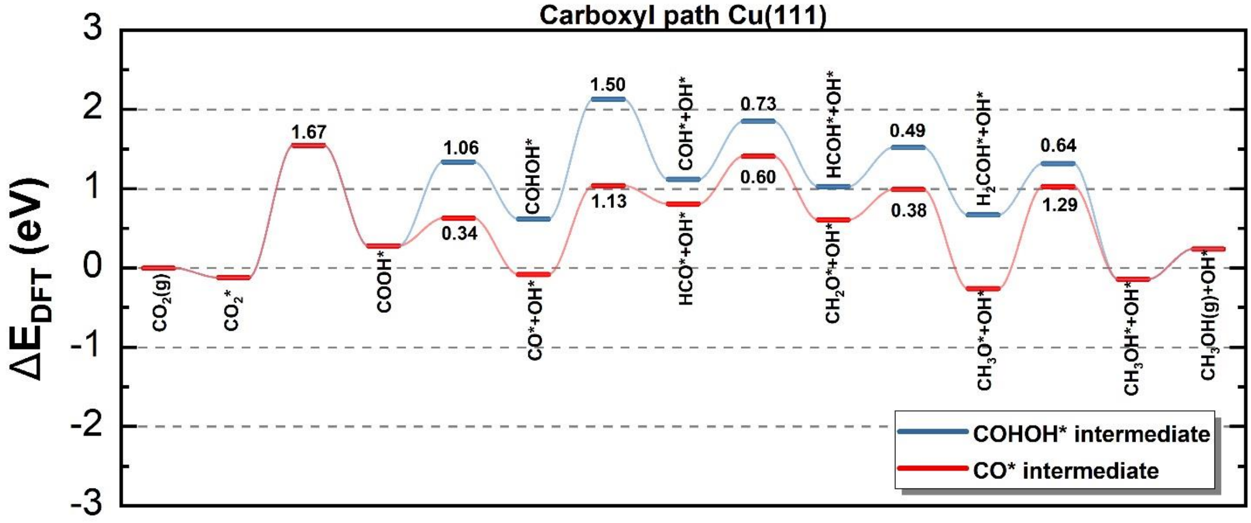

Figure S10: Potential energy diagram of both variants of the carboxyl pathway on Cu(111), the path through COHOH* is indicated in blue, the path via CO* in red. Activation energy barriers are in eV and intermediate states are depicted in the figure.

Both variants of the carboxyl pathway on Cu(111) are depicted in Figure S10. From this figure, it is again clear that the comparison between both variants of the carboxyl pathway for Cu(111) is the same as for Cu(211). The most favourable carboxyl pathway and rate-controlling step are the same for both facets. The barriers for the most favourable carboxyl pathway, i.e., through CO*, on Cu(111) are higher or similar to the barriers on Cu(211), with a rate-controlling step that is 0.18 eV lower on Cu(211). For the pathway through COHOH*, the comparison is less unambiguous. E.g., the barrier for COHOH* dissociation is 0.16 eV higher on Cu(211), while the barrier for HCOH* hydrogenation is 0.08 eV lower on Cu(211).

S.6.3 CO_2 dissociation pathway

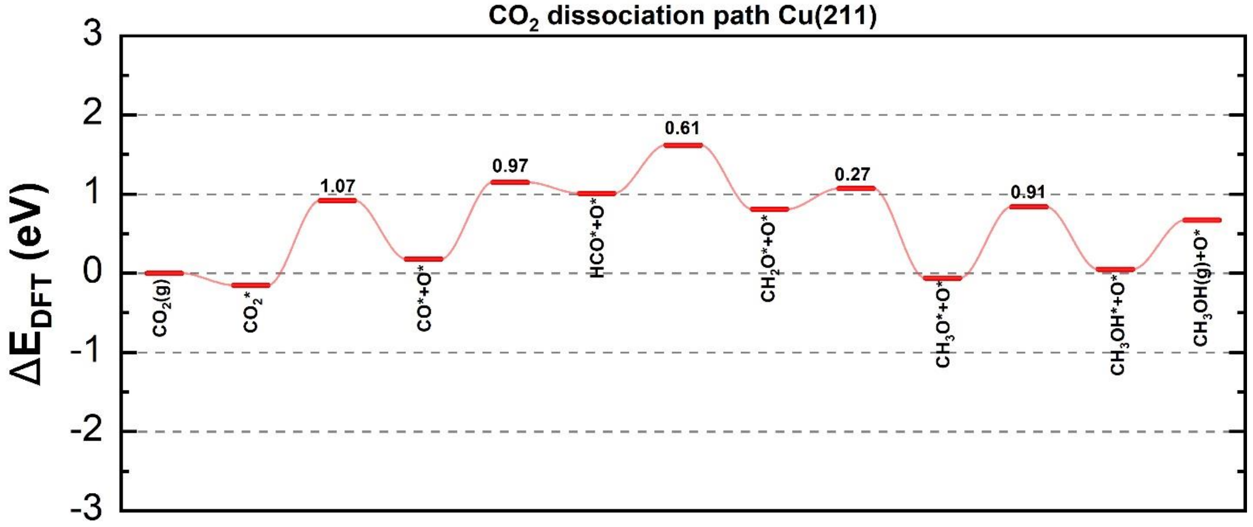

Figure S11: Potential energy diagram of the CO_2 dissociation path on Cu(211). Activation energy barriers are in eV and intermediate states are depicted in the figure.

From the presence of CO* in the carboxyl pathway it becomes clear that the exploration of the CO_2 dissociation pathway, the last pathway discussed in the introduction of the main paper, is important. This pathway is depicted in Figure S11 for Cu(211) and in Figure S12 for Cu(111). The potential energy diagram for Cu(211) highlights that the direct dissociation of adsorbed CO_2* to yield CO* is thermodynamically unfavourable, with a reaction energy of 0.18 eV, and is associated with an energy barrier of 1.07 eV. Subsequently, CO* undergoes hydrogenation to produce CH_3OH via the same reactions as in the carboxyl pathway. The highest barrier and rate-controlling step is CO_2* dissociation with a barrier of 1.07 eV. This pathway thus resembles the carboxyl pathway and can be summarized as CO_2* → CO* → HCO* → CH_2O* → CH_3O* → CH_3OH*.

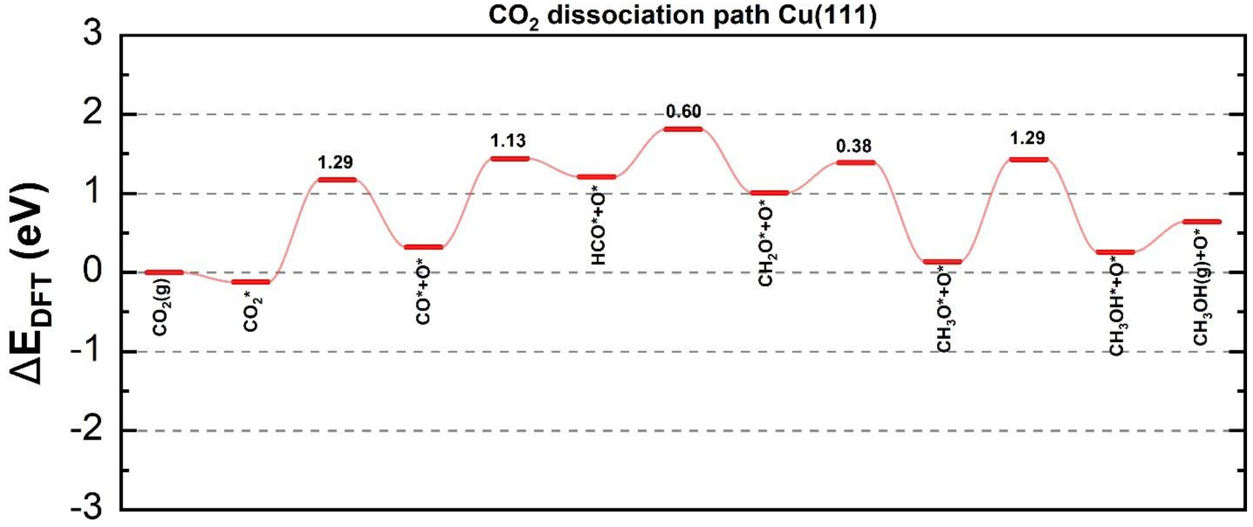

Figure S12: Potential energy diagram of the direct CO_2 dissociation path on Cu(111). Activation energy barriers are in eV and intermediate states are depicted on the figure.

The CO_2* dissociation barrier on the flat Cu(111) surface is 0.22 eV higher than the dissociation barrier on the stepped Cu(211) surface. In previous experimental studies, the CO_2 dissociation barrier on the Cu(110) surface was found to be 0.69 eV[5], while on the relatively flat Cu(100) surface, the barrier was 0.96 eV[6]. The dissociation of CO_2 on stepped and kinked surfaces is significantly easier on stepped surfaces than on flat surfaces, which is consistent with our calculations. The highest barriers on Cu(111) are those for CO_2* dissociation and CH_3O* hydrogenation, both being 1.29 eV. The CO_2 dissociation pathway on Cu(111) is thus the same as the one on Cu(211) but the reactions on Cu(111) have higher barriers. The rate-controlling steps on Cu(111) are CH_3O* hydrogenation and CO_2* dissociation with a barrier of 1.29 eV, which is 0.22 eV higher than the rate-controlling step on Cu(111).

S.6.4 Comparison pathways on Cu(211) and Cu(111)

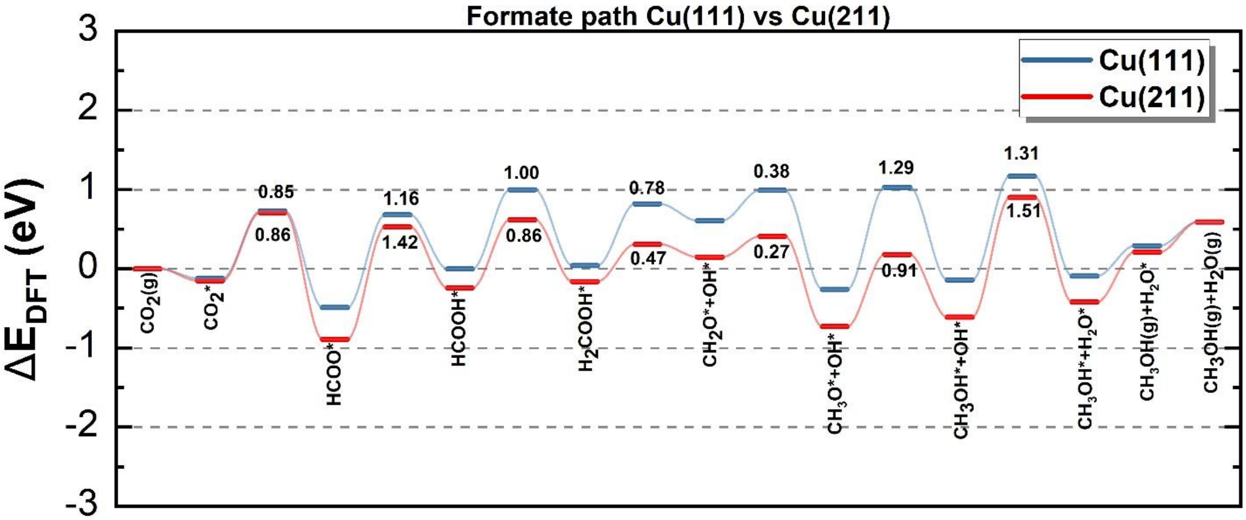

Figure S13: Potential energy diagram of the formate path on Cu(211), in red, and Cu(111), in blue . Activation energy barriers are in eV and intermediate states are depicted in the figure.

References

(1) *Computational Chemistry Comparison and Benchmark Database*. NIST Standard Reference Database 101. http://cccbdb.nist.gov/ (accessed 2023-12-07).

(2) Brosseau, R.; Brustein, M. R.; Ellis, T. H. Water Adsorption on Cu(100): The Effect of Defects. *Surf. Sci.* **1993**, *294* (3), 243–250. DOI: 10.1016/0039-6028(93)90111-V.

(3) Radnik, J.; Ernst, H.-J. Adsorption Geometries of CO on Cu (211). *J. Phys. Chem.* **1999**, *110* (21), 10522–10525. DOI: 10.1063/1.478983.

(4) Schreiner, P. R.; Reisenauer, H. P. Spectroscopic Identification of Dihydroxycarbene. *Angew. Chem. Int. Ed.* **2008**, *120* (37), 7179–7182. DOI: 10.1002/ange.200802105.

(5) Nakamura, J.; Rodriguez, J. A.; Campbell, C. T. Does CO2 Dissociatively Adsorb on Cu Surfaces? *J. Phys.: Condens. Matter* **1989**, *1* (SB), SB149. DOI: 10.1088/0953-8984/1/SB/026.

(6) Rasmussen, P. B.; Taylor, P. A.; Chorkendorff, I. The Interaction of Carbon Dioxide with Cu(100). *Surf. Sci.* **1992**, *269–270*, 352–359. DOI: 10.1016/0039-6028(92)91274-F.
